# ACE2 and Furin Expressions in Oral Epithelial Cells Possibly Facilitate COVID-19 Infection via Respiratory and Fecal–Oral Routes

**DOI:** 10.3389/fmed.2020.580796

**Published:** 2020-12-10

**Authors:** Mei Zhong, Bingpeng Lin, Janak L. Pathak, Hongbin Gao, Andrew J. Young, Xinhong Wang, Chang Liu, Kaibin Wu, Mingxiao Liu, Jian-ming Chen, Jiangyong Huang, Learn-Han Lee, Cui-ling Qi, Linhu Ge, Lijing Wang

**Affiliations:** ^1^Guangzhou Key Laboratory of Basic and Applied Research of Oral Regenerative Medicine, Affiliated Stomatology Hospital of Guangzhou Medical University, Guangzhou, China; ^2^Institute of Oral Disease, Guangzhou Medical University, Guangzhou, China; ^3^Key Laboratory of Guangdong Laboratory Animals, Guangdong Laboratory Animals Monitoring Institute, Guangzhou, China; ^4^Columbus Inpatient Care, Grove City, OH, United States; ^5^Stomatology School of Ji'nan University, Guangzhou, China; ^6^Novel Bacteria and Drug Discovery Research Group (NBDD), Microbiome and Bioresource Research Strength (MBRS), Jeffrey Cheah School of Medicine and Health Sciences, Monash University Malaysia, Bandar Sunway, Malaysia; ^7^School of Life Science and Biopharmaceutics, Vascular Biology Research Institute, Guangdong Pharmaceutical University, Guangzhou, China

**Keywords:** COVID-19, SARS-CoV-2, ACE2 (angiotensin converting enzyme-2), Furin, oral mucosa, single cell RNA seq

## Abstract

**Background:** Coronavirus disease 2019 (COVID-19) is caused by severe acute respiratory syndrome coronavirus 2 (SARS-CoV-2) that mainly transfers from human to human via respiratory and gastrointestinal routes. The S-glycoprotein in the virus is the key factor for the entry of SARS-CoV-2 into the cell, which contains two functional domains: S1 is an angiotensin-converting enzyme 2 (ACE2) receptor binding domain, and S2 is necessary for fusion of the coronavirus and cell membranes. Moreover, it has been reported that ACE2 is likely to be the receptor for SARS-CoV-2. In addition, mRNA level expression of Furin enzyme and ACE2 receptor had been reported in airway epithelia, cardiac tissue, and enteric canals. However, the expression patterns of ACE2 and Furin in different cell types of oral tissues are still unclear.

**Methods:** In order to investigate the potential infective channel of the new coronavirus via the oropharyngeal cavity, we analyze the expression of ACE2 and Furin in human oral mucosa using the public single-cell sequence datasets. Furthermore, immunohistochemistry was performed in mucosal tissue from different oral anatomical sites to confirm the expression of ACE2 and Furin at the protein level.

**Results:** The bioinformatics results indicated the differential expression of ACE2 and Furin on epithelial cells from different oral anatomical sites. Immunohistochemistry results revealed that both the ACE2-positive and Furin-positive cells in the target tissues were mainly positioned in the epithelial layers, partly expressed in fibroblasts, further confirming the bioinformatics results.

**Conclusions:** Based on these findings, we speculated that SARS-CoV-2 could invade oral mucosal cells through two possible routes: binding to the ACE2 receptor and fusion with cell membrane activated by Furin protease. Our results indicated that oral mucosa tissues are susceptible to SARS-CoV-2 that could facilitate COVID-19 infection via respiratory and fecal–oral routes.

## Introduction

Severe acute respiratory syndrome coronavirus 2 (SARS-CoV-2) causes coronavirus disease 2019 (COVID-19) pandemic and mainly triggers acute respiratory distress syndrome (ARDS) and viral sepsis that bring challenges to the patient's treatment (1, 2). The pandemic infection of COVID-19 confirmed nearly 40,000,000 human cases, including >1,100,000 deaths, and the infection rate is continuously increasing worldwide. The present outbreak of COVID-19 has been labeled as a global pandemic by WHO and has posed critical challenges for public health, research, and medical communities (3). Airborne transmission has been recently reported as a dominant route for COVID-19 infection (4). Similarly, many research groups had reported gastrointestinal manifestation of COVID-19 purposing the fecal–oral route as an alternative route of SARS-CoV-2 transmission (5–8). These findings suggest that the epithelial cells of the oropharyngeal cavity provide the binding site to enter and propagate the SARS-CoV-2. However, the expression of possible receptors of the SARS-CoV-2 in different cell types of the oral cavity has not been investigated yet.

Like patients with SARS and Middle East respiratory syndrome (MERS), the clinical manifestations of COVID-19 at illness onset are fever, dry cough, and myalgia. The patients suffer from dyspnea, shortness of breath, respiratory failure, and even death in later stages (2, 9, 10). Besides, COVID-19 patients show oral manifestations, including oral pain, gingivitis, and ulcers, especially for severe cases (11). Dry mouth and amblygeustia are experienced by a relatively high proportion of COVID-19 patients (12). Also, viral enanthema in the oral mucosa is considered as a possible diagnostic challenge in the COVID-19 pandemic (13). As the oropharyngeal cavity is a route of COVID-19 transmission, it might have adverse impacts on patients' oral health. Since COVID-19 seems to stay longer in our society, the insights in the relationship between oral health and COVID-19 could provide crucial information for decision-making in managing this notorious infectious disease.

SARS-CoV-2 entry into the cell is induced by the binding of viral spike protein to host cellular angiotensin-converting enzyme 2 (ACE2) receptors (14, 15). Previous findings indicated that ACE2 plays an essential role in SARS-CoV-2 entry in the host cells. Thus, ACE2-expressing cells may act as target cells and are susceptible to COVID-19 infection (2, 14–18). Hamming et al. (19) had reported the ACE2 receptor expression in various human tissues, including epithelium of the lung and small intestines. As a co-expressed membrane endopeptidase of the ACE2 receptor, Furin has the potential to cleave the viral envelop glycoprotein, thereby enhancing the viral fusion with host cell membranes (20). Genomic characterization of SARS-CoV-2 has revealed that the Furin enzyme could activate the specific site of its spike protein (21). Meanwhile, Li et al. (22) showed that a putative Furin cleavage site in the spike protein of SARS-CoV-2 facilitates the virus–cell fusion. The Furin cleavage site is absent in SARS-CoV. The presence of the Furin cleavage site in SARS-CoV-2 showed distinct clinical symptoms (23, 24). Furthermore, online single-cell sequence datasets analysis unveiled that Furin was expressed in coronavirus's potential target organs, such as lung, heart, nose, rectum, colon, intestine, and ileum (22). Hence, the expression and distribution of ACE2 and Furin in the epithelium of the oral cavity could be critical for the coronavirus invasion to the host cells. However, information on the expression and distribution of ACE2 and Furin in the human oral cavity is limited.

Single-cell RNA sequencing (scRNA-Seq) technique provides an avenue to understand gene regulation networks and the complexity of cell-to-cell heterogeneity at the single-cell level. scRNA-Seq has diverse applications on bioinformatics, stem cell differentiation, organ development, whole-tissue subtyping, and tumor biology (25–27). scRNA-Seq captures cellular differences underlying tumor biology at a higher resolution than regular bulk RNA-Seq and has revolutionized studies of cancer mechanisms and personalized medicine (28). For a better understanding of the potential COVID-19 infection risk in the oral cavity, we explored whether ACE2 and Furin are expressed and the composition and proportion of their expressing cell in different oral tissues based on the public scRNA-seq profiles from Gene Expression Omnibus (GEO) public databases. Furthermore, we performed immunohistochemistry analysis for ACE2 and Furin in human oral epithelium tissue sections. Oral epithelium showed the expression of ACE2 and Furin in both mRNA and protein levels, indicating the oral cavity as a platform for COVID-19 invasion via the respiratory route and possibly fecal–oral route.

## Materials and Methods

### Public Single-Cell RNA Sequencing Dataset Acquisition

scRNA-Seq datasets (GSE103322), including the gene expression and cell type annotation from human oral squamous cell carcinoma (OSCC) tissue, were downloaded from the GEO database (https://www.ncbi.nlm.nih.gov/geo/). Patient characteristics and demographics for the dataset used are listed in Supplementary Table 1.

### Single-Cell Sequencing Analysis

scRNA-Seq datasets (GSE103322) were downloaded from the GEO database. Datasets of five patients, which have the closest histological features with normal tissue, were selected for further analysis. The scRNA-Seq datasets of GSE103322_HN_UMI_table.txt were used to subsequently analyze based on R package Seurat (Version 3.6.2). For quality control, we removed cells with <50 genes, as well as the cells with mitochondrial content higher than 5%. Besides, the genes detected in <3 cells were filtered out in the function Create Seurat Object. Subsequently, the data were log-normalized using the function Normalize Data with the default parameters. The Find Variable Genes function was used to determine the highly expressed variable genes, followed by principal component analysis (PCA) dimensionality reduction by RunPCA function. PCA components, which *P* < 0.05, were selected to analyze further using two-dimensional t-distributed stochastic neighbor embedding (tSNE). The setting of k.param was 20 in the function Find Neighbors, and the setting of the resolution was 0.5 in the function Find Clusters. Finally, the cell types were assigned based on their canonical markers. The functions of DotPlot and VlnPlot displayed the gene expression of different cell types. The significant level was set as 0.05.

### Human Oral Tissue Specimens

Twelve normal tissues of the oral mucosa (three buccal and gingival tissues, two lip, tongue, and palatal tissues, respectively) were taken from 12 different patients with clinically diagnosed fibrous epithelial polyp or benign tumor (19, 29), whose average age was 53 years. The healthy oral mucosa tissue was collected from a 2-cm distance of the tumor edge. Patient exclusion criteria of this study were patients with a history of smoking, periodontitis, uncompensated diabetes mellitus, immunocompromised status, under radiotherapy and/or chemotherapy, and systemic diseases. Vulnerable populations such as pregnant women, minors (i.e., under 18 years old), and seniors (i.e., over 60 years old) were also excluded. Written informed consent was obtained for each participant, and the study was in accordance with the Declaration of Helsinki and the guidelines and approved by the Clinical Ethics Committee of Affiliated Stomatology Hospital of Guangzhou Medical University (Approval no. KY2020002). Tissue morphology was evaluated in hematoxylin and eosin-stained sections by a qualified pathologist.

### Immunohistochemical Staining

The selected tissues were fixed in 10% neutral formalin solution and embedded in paraffin. The paraffin sections (4 μm in thickness) were deparaffinized in xylene, rehydrated in a graded series of alcohol. Human lung bronchi tissue sections were used as positive control of ACE2 and Furin expression. The tissue sections were then incubated in citrate buffer (pH 6.0) for 5 min at 120°C, and the endogenous peroxidase was blocked by 0.3% H_2_O_2_ for 10 min. Subsequently, the tissue sections were dried and incubated with Rabbit Anti-ACE2 antibody (cat. no. ab15348, Abcam, Cambridge, CB, UK) antibody, Anti-Furin antibody (cat. no. bs-13228R) overnight at 4°C. The negative control sections were incubated with 1% rabbit serum containing phosphate buffered saline (PBS) instead of the primary antibody. The next day, the tissue sections were incubated with the horseradish peroxidase (HRP) (Goat Anti-Rabbit IgG H&L)-conjugated secondary antibody for 1 h at room temperature. Peroxidase activity was developed by using 3,3′-diaminobenzidine (DAB) for 8 min and counterstained with hematoxylin. A qualified pathologist analyzed the staining. The ACE2 and Furin staining intensities were semiquantitatively graded according to the previously described method (30).

## Results

### Identification of Cell Types in the Oral Mucosal Tissues

**Figure 1:**
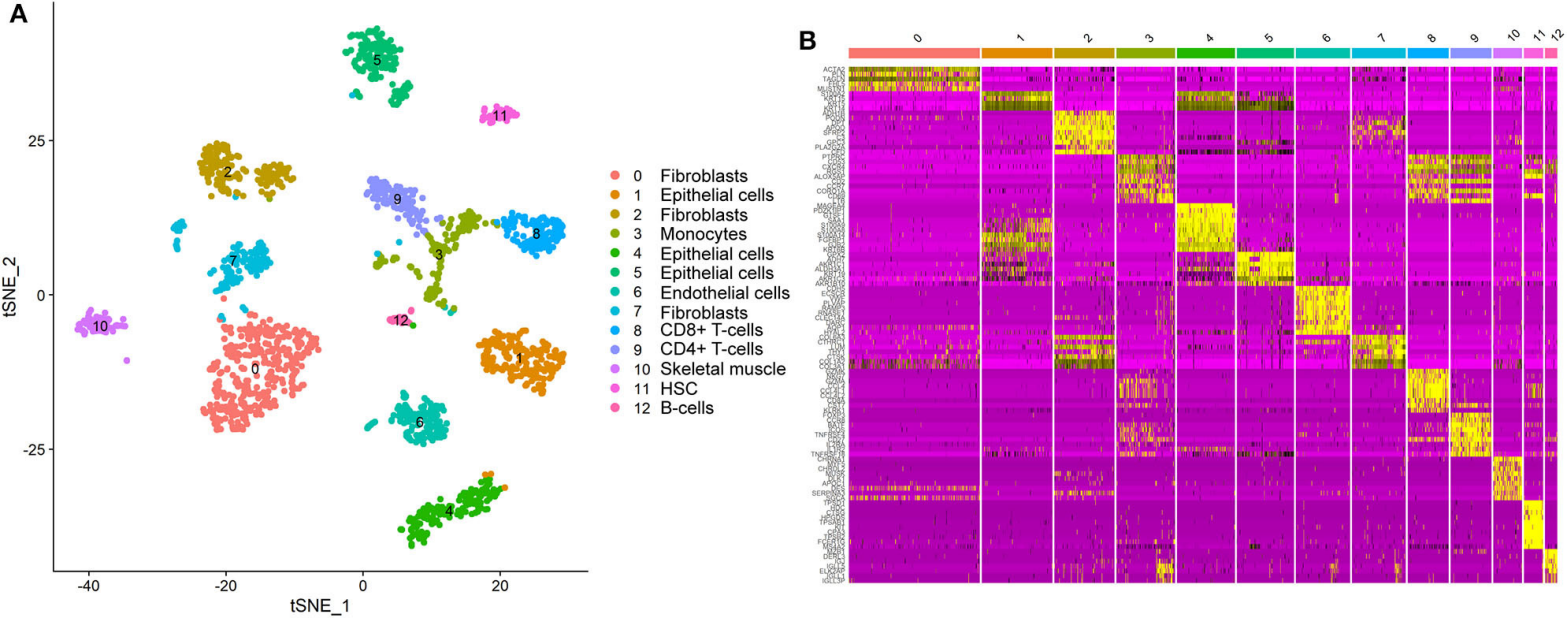
Single-cell atlas of the human oral mucosa. **(A,B)** Analysis of single-cell sequencing data identified 13 cell subclusters in the oral mucosal cells. Thirteen distinct cell clusters existed in the oral tissues **(A)**, including epithelial cells, fibroblasts, T cells, and B cells. The heatmap of the main corresponding cell marker gene expression profiles across the cell types is plotted in **(B)**. The marker gene of epithelial cells included KRT6B, GJB2, SFN, and KRT14.

We detected the ACE2-expressing cell types in oral tissue by analyzing the scRNA-Seq data. A total of 1,843 individual cells from the oral mucosa of five patients were analyzed. Using the unsupervised graph-based clustering, we found that at least 13 distinct cell clusters existed in the oral tissues (Figure 1A), including epithelial cells (clusters 1, 4, 5), fibroblasts (clusters 0, 2, 7), T-cells (clusters 8, 9), and B cells (cluster 12). The heatmap of the main corresponding cell marker gene expression profiles across the cell types can be found in Figure 1B. The marker gene of epithelial cells included KRT6B, GJB2, SFN, and KRT14; fibroblasts included THY1, COL1A1, DCN, and ACTA2; B cells included SLAMF7, CD19, CD20, and CD79A; T cells included CD2, CD3, and CD45.

### Identification of Angiotensin-Converting Enzyme 2-Expressing Cells in the Oral Mucosa

**Figure 2:**
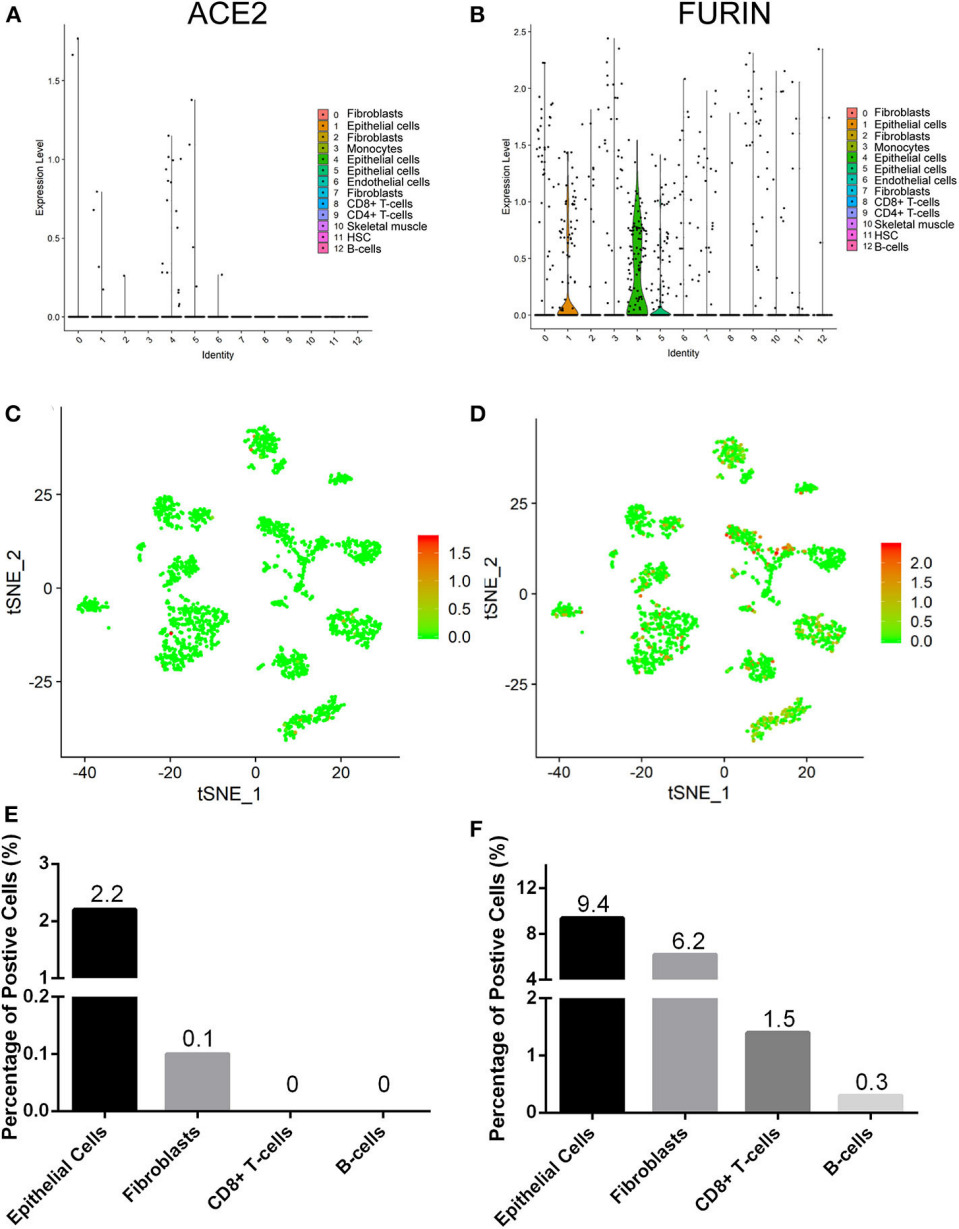
The expression profiles of angiotensin-converting enzyme 2 (ACE2) **(A,C,E)** and Furin **(B,D,F)** in the oral mucosal cells. Violin and scatter plot showed the expression profile of the ACE2 receptor **(A,C)** and Furin **(B,D)** in oral mucosal cells. ACE2 and Furin were mainly expressed in epithelial cells. **(E,F)** The percentage of ACE2- and Furin-positive cells in the oral mucosa.

We explored the online datasets to find out the expression level of ACE2 in the oral mucosa. A single-cell atlas from analysis of scRNA-Seq data of the human oral mucosa identified 13 cell subclusters in the oral mucosal cells, including epithelial cells, fibroblasts, T cells, and B cells (Figure 1). The clusters 1, 4, 5, and 6 are epithelial cell clusters. The red dots in the scatter plot (Figures 2C,D) showed the expression profiles of ACE2 and Furin in the 13 distinct cell clusters identified from Figure 1A. As expected, data from GEO indicated that ACE2 receptor expression level was relatively higher in epithelial cells (clusters 1, 4, 5) (Figures 2A,C), while very little cellular expression of ACE2 was found in fibroblasts (cluster 2). Then, we calculated the percentage of ACE2-expressing cells in the dataset (Figure 2E). We find that, in oral tissue, the percentage of ACE2-positive cells was 2.2%. Among them, 92% were epithelial cells. The negative control of oral tissue immunohistochemistry (IHC) is listed is Supplementary Figure 1.

### Identification of Furin-Expressing Cells in the Oral Mucosa

To determine the cell types of expressing Furin, we analyzed the Furin mRNA expression using tSNE method. Violin and scatter plot showed that Furin was highly expressed in epithelial cells, followed by fibroblasts, T cells, and endothelial cells of the oral mucosal tissues, but hardly expressed in B cells (Figures 2B,D). Meanwhile, the proportion of Furin expressing cells was ~10%. Among them, epithelial cells make up more than 55% (Figure 2F).

### Angiotensin-Converting Enzyme 2 Protein Is Highly Expressed in the Epithelial Layer of the Buccal Mucosa, Lip, Palate, Tongue, and Gingiva

**Figure 3:**
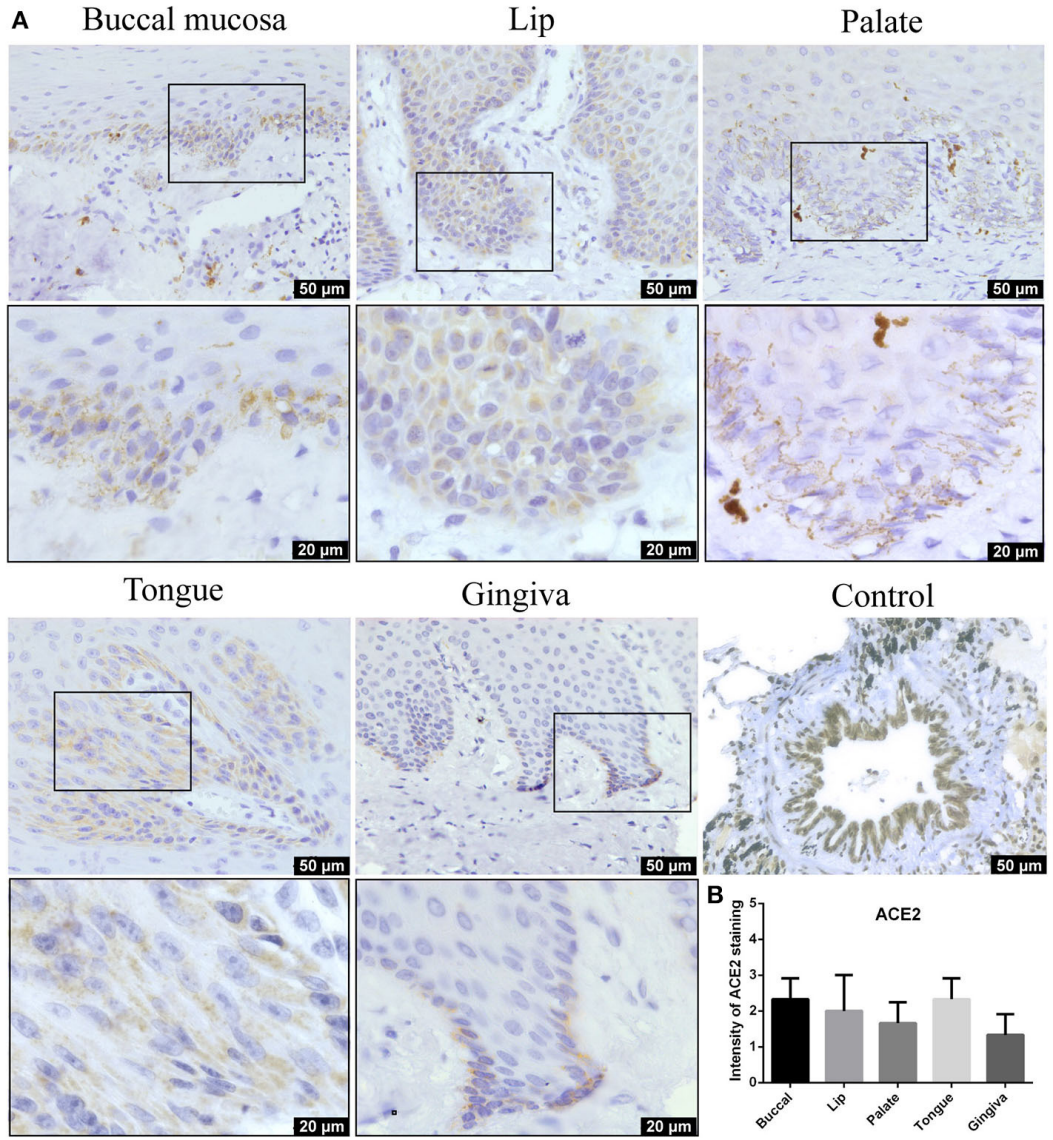
**(A)** Representative immunohistochemistry (IHC) images of oral tissue showing the expression of angiotensin-converting enzyme 2 (ACE2) protein in buccal, lip, palate, tongue, and gingival mucosa and lung bronchi (positive control). **(B)** Semiquantitative analysis of IHC intensity of ACE2 staining for various oral tissues. Data are presented as mean ± standard deviation (SD).

IHC analysis further determined the protein level expression of ACE2 in the oral mucosa. ACE2 and Furin were highly expressed in human lung bronchi tissue sections, indicating the specificity of the primary antibodies used in this study. The first remarkable finding was that ACE2 was expressed in epithelial cells in all the tissues studied (Figure 3). Results indicated that the expression level of ACE2 protein was significantly higher in the lip, tongue, and buccal mucosa, especially the epithelial cells in the basal layer, although the mRNA expression level is not as high (Figure 2). The ACE2-positive cells in the gingival and palatal tissues were limited. The corresponding gingiva and palate epithelial cells showed weak positive ACE2 staining. Systematic ACE2 expression levels in different oral sites are shown in **Figure 5**. Also, we found that ACE2 was expressed in fibroblasts and endothelial cells. These results indicate oral epithelial cells as the potential targets of SARS-CoV-2.

### Furin Protein Was Highly Expressed in the Normal Oral Mucosa

**Figure 4:**
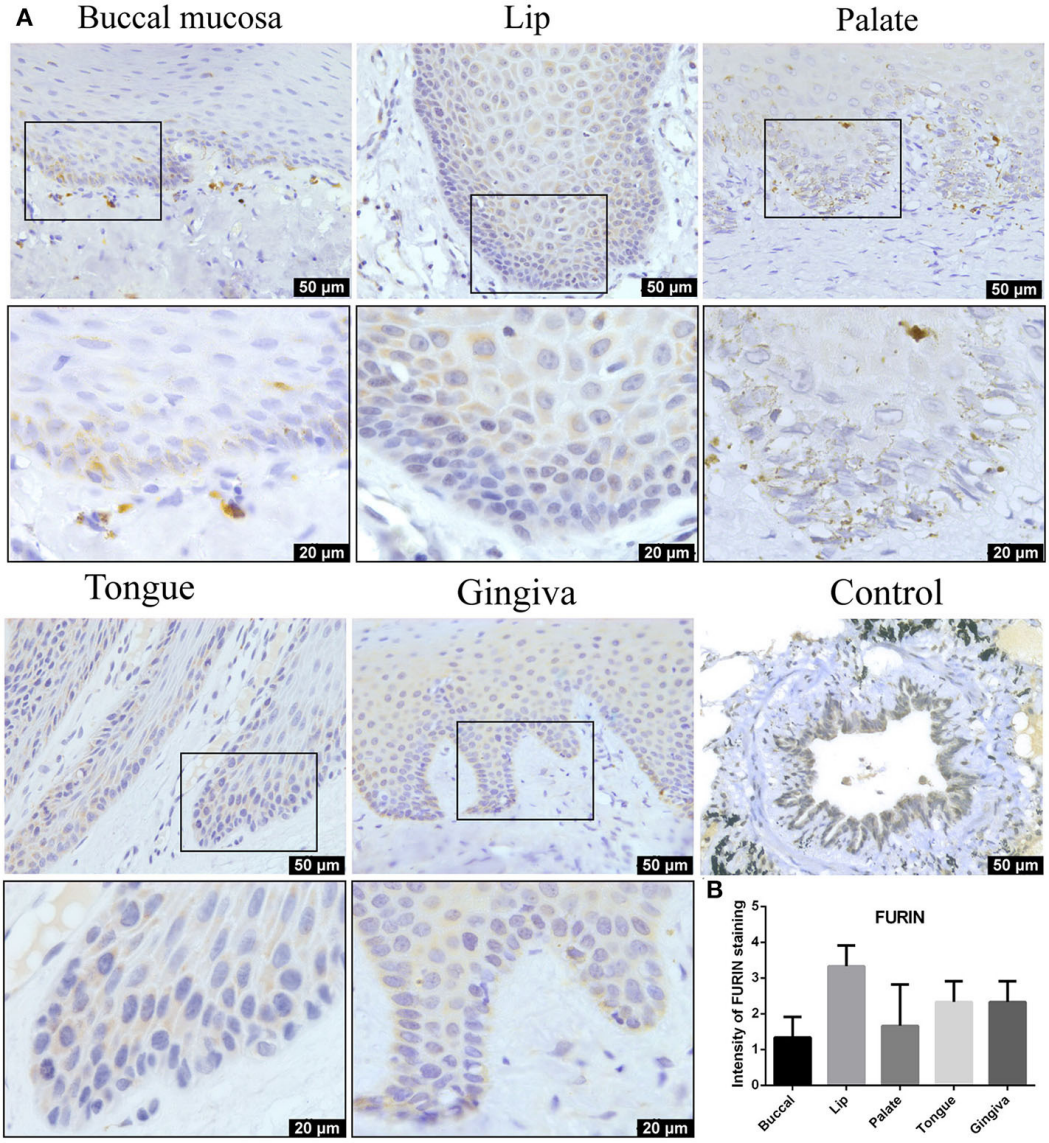
**(A)** Representative immunohistochemistry (IHC) images of oral tissue showing the expression of Furin protein in buccal, lip, palate, tongue, and gingival mucosa and lung bronchi (positive control). **(B)** Semiquantitative analysis of IHC intensity of Furin staining for various oral tissues. Data are presented as mean ± SD.

**Figure 5:**
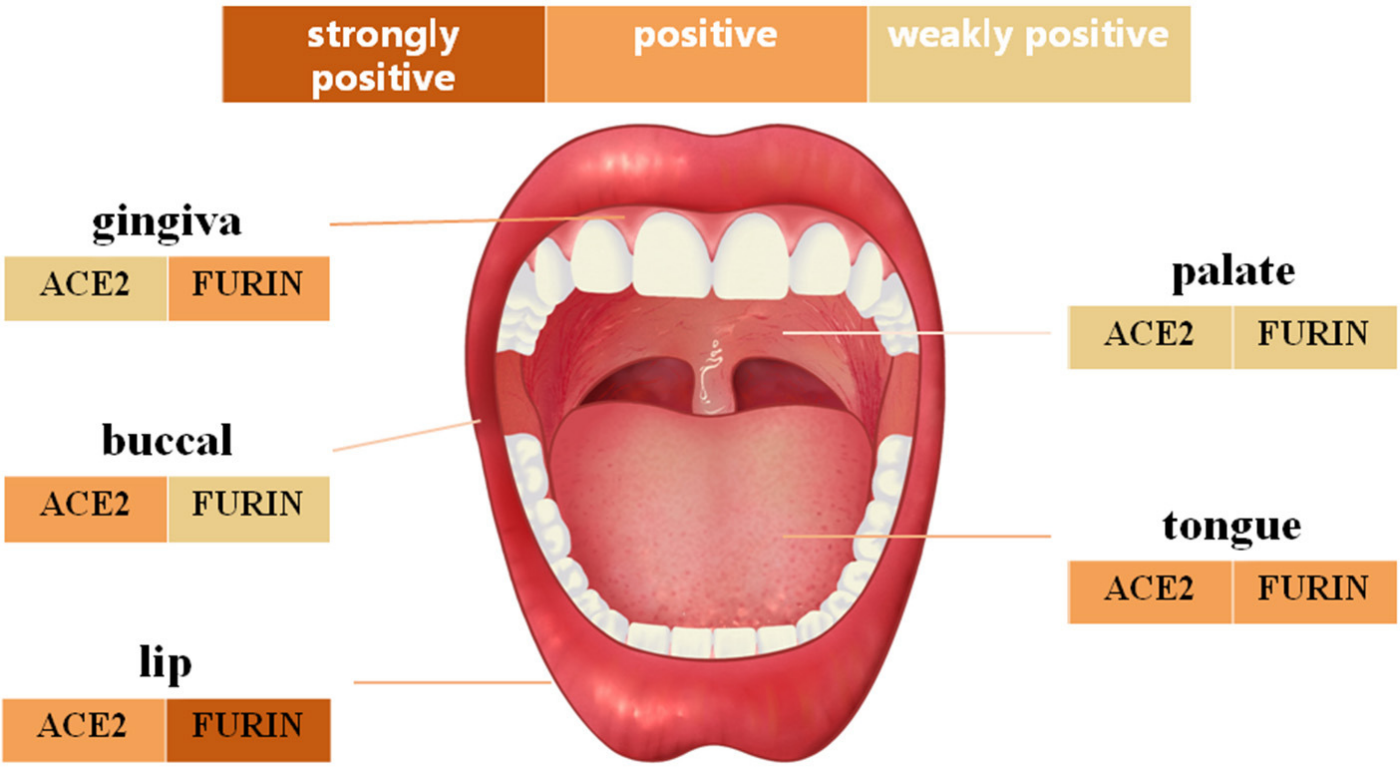
The schematic diagram of angiotensin-converting enzyme 2 (ACE2) and Furin protein expression in different oral anatomical sites.

Furin mRNA expression has been described mainly in oral epithelial cells through scRNA-Seq technique. To determine whether this differential expression of Furin was maintained at the protein level, oral tissues from different sites were screened. As shown in Figure 4, moderate to mild immunostaining in the cytoplasm of epithelial cells was observed in five normal oral tissues. Surprisingly, except for the positive cells in the basal layer of the oral epithelium, the spinous layer in all examined tissues also turned out with large numbers of Furin-positive cells. In addition, the percentage of Furin-positive cells in the lip, tongue, and gingiva was higher than that of buccal and palatal mucosa. Systematic Furin expression levels in different oral sites are shown in Figure 5.

## Discussion

Highly infectious nature and pandemic spreading of COVID-19 overwhelmed the public health services in the world and have become a global public health problem (31). Studies had reported both the respiratory and gastrointestinal tract manifestations of COVID-19 (4, 32). As mentioned before, the ACE2 receptor in the host cell membrane and Furin cleavage site of SARS-CoV-2 are the key factors that allowed the virus to invade the host cells (22, 23). In this study, we found the expression of ACE2 and Furin in oral epithelial cells in both mRNA and protein levels. Since the oral cavity is connected to both the respiratory and gastrointestinal tract, the ACE2 and Furin in oral mucosal cells could be the possible facilitators of COVID-19 infection *via* respiratory and fecal–oral routes.

Many studies have shown that the ACE2 receptor was highly expressed in respiratory epithelium, kidney, testis, digestive system, and cardiovascular system (6, 18, 33). Pieces of literature using bulk RNA sequencing data found that ACE2 is expressed in oral tissues, which could not accurately reflect the expression of ACE2 in the single-cell level (29, 34). Chen et al. benefited from the single-cell sequencing data to further prove the expression of ACE2 receptor in the tongue, buccal mucosa, and gingiva (14, 19, 34). However, these studies were based on public databases and did not further analyze the protein level expression. In the current study, we firstly analyzed the ACE2 expression in different cell types present in various oral anatomical sites using the scRNA-Seq public data from five human subjects. We further confirmed the protein level expression of ACE2 in histological tissue sections from different anatomical sites of 12 human subjects by IHC. Results indicated that ACE2 is mainly expressed in epithelial cells in all included oral tissues, especially in the buccal mucosa, lip, and tongue. Our results demonstrated that compared with other oral anatomical sites, buccal mucosa, lip, and tongue showed a higher expression of ACE2, suggesting that these sites are more susceptible to SARS-CoV-2-invasion.

Team of virologists from Cornell University reported another structural analysis of the coronavirus S protein, suggesting that the Furin cutting site allows the SARS-CoV-2 virus to enter cells in a very different way than SARS, and this may regulate the virus stability and infection (35). Other research teams have also identified this activation site, suggesting that it may enable the virus to spread effectively between humans (21). It has been demonstrated that SARS-CoV-2 could fuse with the cell membrane and then gain entry into the target cells (36, 37). Researchers have found Furin proteases in many tissues, including the lungs, liver, and small intestine (23). However, limited reports were seen regarding the expression of Furin in the oral cavity, and whether the novel coronavirus could spread by the oral cavity is not fully understood. Thus, we reported both gene and protein level expression patterns of Furin enzyme in epithelial cells of five different oral anatomical sites. As expected, Furin was found abundantly enriched in oral mucosa cells, particularly in epithelial cells, and the proportion of Furin-positive cells was higher than that of ACE2-positive cells. Our results would be valuable for the prevention and management of the COVID-19.

Previous reports had demonstrated that Furin protein was expressed in metastasizing OSCCs (38). Besides, Furin was expressed in normal epithelium and upregulated in SCCs from three different organs (30). But the reports of Furin protein expression in the healthy oral cavity are limited. For further validating the results in the gene level, Furin IHC was therefore performed on conventional sections for the first time to assess its expression in oral mucosa obtained from five different sites. It is noteworthy that moderate positive expression of Furin protein was observed in tongue, gingiva, and lip, while weakly positive in the buccal and palatal tissues. Shreds of data from the literature indicate that SARS-CoV-2 enters host cells by binding to ACE2, and the viral S protein is cleaved by transmembrane protease serine 2 (TMPRSS2) (39). Furin cleaves S1/S2 site during the process of S protein transport and virus assembly (40). As a result, we speculated that SARS-CoV-2 could attach to the cell membrane of the oral mucosal cells, then enter into the host cells via the ACE2 and Furin activity. Therefore, Furin could be a possible target to reduce COVID-19 infection.

For entry of SARS-CoV-2 to host cell, viral surface glycoprotein spike (S) must be cleaved at two different sites (S1/S2 and S2) by host cell proteases (41). The S can be cleaved by the Furin at the S1/S2 site and TMPRSS2 at the S2′ site (42). The spread of SARS-CoV-2 also depends on TMPRSS2 activity (43). The Furin-mediated precleavage at the S1/S2 site in infected cells might promote subsequent TMPRSS2-dependent entry into target cells. TMPRSS2 is essential for SARS-CoV-2 activation and multiplication in airway epithelial cells. A combination of TMPRSS2 inhibitor and Furin inhibitor more potently inhibits SARS-CoV-2 replication in human airway epithelial cells compared to any single inhibitor (42). This indicates the important role of Furin and TMPRSS2 proteases in facilitating the entry of SARS-CoV-2 in host cells. The expression of TMPRSS2 in oral tissues should be further investigated.

Strikingly, the scRNA-Seq data analysis and IHC results indicated that ACE2 and Furin were both abundantly expressed in oral mucosa, especially on oral epithelial cells. In other words, two key proteins of SARS-CoV-2 entering the target cell in different routes are both present in oral mucosa, which implied the potential attack and spread of SARS-CoV-2 in the oral tissues. Moreover, the live SARS-CoV-2 was consistently detected in the self-collected saliva of 91.7% (11/12) COVID-19 patients (44). It could be transmitted via saliva, directly or indirectly, even among asymptomatic infected patients. The existence of SARS-CoV-2 in nasal and throats swab samples suggests the strong spreadability of COVID-19 via the oral cavity (45). Thus, it is suggested that all healthcare workers should focus on taking effective measures to prevent the virus from spreading via the oral cavity, and potential impairment of oral tissues should be evaluated after infection with SARS-CoV-2, giving more evidence to the prevention and treatment of COVID-19. Strict precautions should be taken to protect from direct exposure of oral tissues to SARS-CoV-2 contamination.

## Conclusions

Our results systematically investigated the expression profiles of Furin enzyme and ACE2 receptor in the oral cavity at the gene and protein levels for the first time. Furthermore, significant expression of Furin and ACE2 was discovered in oral epithelial cells, implying the possibility of COVID-19 transmission through the oral mucosa, which provides new insight into the future prevention strategy and clinical care. More evidence is still needed to reinforce the current findings.

## Data Availability Statement

The original contributions presented in the study are included in the article/Supplementary Materials, further inquiries can be directed to the corresponding author/s.

## Ethics Statement

The studies involving human participants were reviewed and approved by Ethics Committee of Stomatology Hospital of Guangzhou Medical University. The patients/participants provided their written informed consent to participate in this study.

## Author Contributions

BL, MZ, JP, HG, LW, and LG contributed to the study design. BL, MZ, and HG contributed to the sample collection. XW, CL, KW, and ML contributed to the IHC and analysis. L-HL, C-lQ, JH, and J-mC contributed to the data collection, analysis, and interpretation. MZ, L-HL, and JP contributed to the manuscript preparation. All authors approved the final version of the manuscript.

## Conflict of Interest

The authors declare that the research was conducted in the absence of any commercial or financial relationships that could be construed as a potential conflict of interest.
