# Supplementary material for: ACE2 and Furin Expressions in Oral Epithelial Cells Possibly Facilitate COVID-19 Infection via Respiratory and Fecal–Oral Routes
Source: Front Med (Lausanne). 2020 Dec 10;7:580796. doi: 10.3389/fmed.2020.580796 (PMC7758442; doi:10.3389/fmed.2020.580796)
Supplement: Supplementary file 1 [file Table_1.pdf]

Supplementary Table 1

Designation	Age/Sex	Primary site	Cancer stage	Grade	LVI
MEP 6	88/F	right floor of mouth	T4aN2c	1	Absent
MEP 8	82/F	right hard palate	T4aN0	1	Absent
MEP 16	63/F	left lateral tongue	T2N0	1	Absent
MEP 22	77/M	left buccal mucosa	T1N0	2	Absent
MEP 28	58/M	right lateral tongue	T2N2c	1	Present

Table 1. Clinical characteristics and demographics of the patients included in dataset study. (LVI = lymphovascular invasion)
