# Supplementary material for: ACE2 and Furin Expressions in Oral Epithelial Cells Possibly Facilitate COVID-19 Infection via Respiratory and Fecal–Oral Routes
Source: Front Med (Lausanne). 2020 Dec 10;7:580796. doi: 10.3389/fmed.2020.580796 (PMC7758442; doi:10.3389/fmed.2020.580796)
Supplement: Supplementary file 2 [file Image_1.pdf]

Supplementary Figure 1

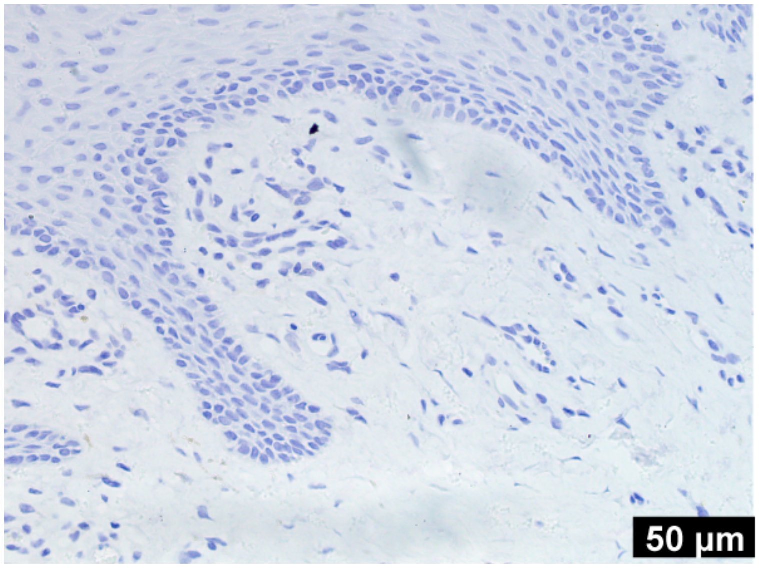

Supplementary Figure 1. Negative control image of oral tissue immunohistochemistry.
